# Staging of mobility, transfer and walking functions of elderly persons based on the codes of the International Classification of Functioning, Disability and Health

**DOI:** 10.1186/1471-2318-13-16

**Published:** 2013-02-15

**Authors:** Jiro Okochi, Tai Takahashi, Kiyoshi Takamuku, Reuben Escorpizo

**Affiliations:** 1 Tatsumanosato Geriatric Health Service Facility, Tatsuma, Daitou, Osaka, Japan; 2 International University of Health and Welfare, Aoyama, Tokyo, Japan; 3 Souseien Geriatric Health Service Facility, Nakatsu, Oita, Japan; 4 Japan Association of Geriatric Health Service Facilities, Shiba, Tokyo, Japan; 5 ICF Research Branch in cooperation with the WHO Collaborating Centre for the Family of International Classification in Germany (DIMDI), Nottwil, Switzerland; 6 Swiss Paraplegic Research, Nottwil, Switzerland; 7 Department of Health Sciences and Health Policy, University of Lucerne, Lucerne, Switzerland; 8 Department of Physical Therapy, Louisiana State University Health Sciences Center, New Orleans, LA, USA

## Abstract

**Background:**

The International Classification of Functioning, Disability and Health (ICF) was introduced by the World Health Organization as a common taxonomy to describe the burden of health conditions. This study focuses on the development of a scale for staging basic mobility and walking functions based on the ICF.

**Methods:**

Thirty-three ICF codes were selected to test their fit to the Rasch model and their location. Of these ICF items, four were used to develop a Guttman- type scale of “basic mobility” and another four to develop a“walking” scale to stage functional performance in the elderly. The content validity and differential item functioning of the scales were assessed. The participants, chosen at random, were Japanese over 65 years old using the services of public long-term care insurance, and whose functional assessments were used for scale development and scale validation.

**Results:**

There were 1164 elderly persons who were eligible for scale development. To stage the functional performance of elderly persons, two Guttman-type scales of “basic mobility” and “walking” were constructed. The order of item difficulty was validated using 3260 elderly persons. There is no differential item functioning about study location, sex and age-group in the newly developed scales. These results suggested the newly developed scales have content validity.

**Conclusions:**

These scales divided functional performance into five stages according to four ICF codes, making the measurements simple and less time-consuming and enable clear descriptions of elderly functioning level. This was achieved by hierarchically rearranging the ICF items and constructing Guttman-type scales according to item difficulty using the Rasch model. In addition, each functional level might require similar resources and therefore enable standardization of care and rehabilitation. Illustrations facilitate the sharing of patient images among health care providers. By using the ICF as a common taxonomy, these scales could be used internationally as assessment scales in geriatric care settings. However these scales require further validity and reliability studies for international application.

## Background

In 2001, the World Health Organization (WHO) approved the International Classification of Functioning, Disability and Health (ICF) to describe functioning in health and health-related contexts. The challenges of implementing the ICF [1] in various fields such as medicine, rehabilitation, long-term care, or social care include the operationalization and quantification of the ICF categories [2]. Unlike the International Classification of Diseases (ICD) [3], also developed by the WHO, to which medical records can serve as an information resource, the ICF measures the problems in an individual’s functioning with respect to a health condition. The ICF provides alphanumeric codes that are arranged in a hierarchy for each ICF category or functioning domain. The number of digits in an ICF code represents an increasing level of precision in the categorization or definition for each function in that domain. However, the high number of codes (n = 1434) makes the use of the ICF by health care professionals particularly challenging. Therefore, to facilitate the use of the ICF codes, it is necessary to tailor them to the target population.

As the ICF was developed as a classification system, it requires an additional step for use as a measurement system, i.e., using a qualifier with the ICF code. A user must select an ICF code, followed by measurement using an ICF qualifier. Qualifiers are numeric codes that specify the extent or the magnitude of the disability in that category. The original ICF qualifier is used to record the severity of the problem: no problem; mild; moderate; severe; or complete problem (included in the codes are qualifier 8 (not specified) and qualifier 9 (not applicable)). However, this approach prohibited us from using the ICF for two reasons. First, it was difficult to select relevant ICF codes from the approximately 1434 ICF codes, and if we selected ICF codes for each person, we could not compare the specific function to other persons, because the ICF codes selected for various individuals may not be the same. Second, the reliability of a qualifier for quantification of severity of a disability was not always satisfactory [4,5].

Therefore, for adaptation of the ICF codes, *a priori* selection of ICF codes specific to a target population can minimize the burden of selecting numerous ICF codes. In addition, the use of a simpler qualification tool makes the ICF easier to use as a basis for measurement

There have been several studies aimed at tailoring the number of ICF codes [4,6-9]. ICF codes related to condition-specific ICF items, such as the codes for osteoarthritis and other chronic conditions, were selected in the development of ICF Core Sets [2,10]. This developmental effort facilitated condition-specific selection of ICF codes, but the ICF codes selected for one chronic condition may not necessarily be adaptable to other chronic conditions. The WHO provided the ICF checklist as a simple version of the ICF; however, the broad and vague definitions of the ICF codes used in the checklist limit its use in a target population such as elderly patients, because some ICF codes do not have high reliability for the intended population [4,5].

An alternative approach is to create linkage between the ICF and existing measures of activities of daily living (ADLs) and health-related quality of life (HRQOL) [2,11]. This approach has allowed ICF users to tailor the number of ICF categories to fit specific clinical needs [12,13]. This approach has qualitatively linked the ICF codes to existing ADL limitation-related scales such as the Functional Independence Measure (FIM) [14]. However, in these cross-linking approaches, the absence of quantitative links limits the use of the ICF for measurement scales.

Some studies have tried to link the existing scale to the ICF codes quantitatively [15-17]. An example of such a linkage has been established between the *Typology of the Aged with Illustrations* (TAI) and the ICF [18,19]. The TAI contains four Guttman-type scales for Mobility, Cognitive functioning, Eating, and Toileting. Each scale includes five thresholds that enable staging of the functioning of elderly persons. For example, the following five items are used as thresholds in the TAI mobility scale: threshold 5, “stair climbing”; threshold 4, “walking short distance”; threshold 3, “moving around on a flat floor”; threshold 2, “transferring, maintaining sitting position” and threshold 1, “rolling over on beds”. A Guttman scale is composed of a set of binary items with yes or no answers, with similar content, but differing in difficulty. In this case, items are arranged in order of difficulty so that an individual who performs a particular item also performs items of a lower difficulty rank-order. However, it has been shown that some items used in a TAI scale are not in the order of difficulty when they are assessed with the Rasch model [19].

Another approach is the proposed functional staging measurement. In this measurement, sets of items are used to construct scores; which are then converted into hierarchical stages using cut-off scores. Functional staging provides a detailed description of an individual’s expected ability within each identified stage, including the types of activities he or she can do. This is achieved by cross-linking Activity Measure for Post-Acute Care (AM-PAC) items to the ICF [5]. However, the items used on the AM-PAC are numerous and are not always linkable to the ICF codes. For example, “Fastening a necklace (clasp) behind your neck” is difficult to code in terms of the ICF.

Therefore, in this study, the authors constructed a Guttman-type scale using the response pattern of the ICF items analyzed by the Rasch model. If we could successfully build such a scale starting from ICF codes, we could obtain a simple scale with a staging property.

The Rasch model assumes that the probability that a person will fit into a category within an item is a logistic function of the difference between the person’s ability (θ) and the difficulty of the item (b) [20]. The probability of success (or failure) of an item or a task is a binary item (such as failure or success in transferring from a bed), and can be expressed as

$$\text{Pi}\left(\theta \right)=\frac{e^{\left(\theta -b_{i}\right)}}{1+e^{\left(\theta -b_{i}\right)}}$$

where Pi (θ) is the probability that respondents with ability θ will answer item i correctly (or be able to do the task specified by that item i). From this formula, the expected pattern of responses to an item set is determined given that estimated θ and b.

If the items with a binary response pattern fit the Rasch model, they provide a Guttman-like response structure. For this purpose, we used a binary-type response for each ICF item in this study. In the Rasch model, the Guttman response pattern is the most probable response pattern for a person when items are ordered from least difficult to most difficult. Using these characteristics of the Rasch model, we used the item fitted to the Rasch model as a threshold item in the Guttman-type scale. Therefore, selected ICF items are used as the thresholds for the boundaries between categories. Using this property of the Rasch model, we constructed two Guttman-type scales that can be used as a staging tool.

The objective of our study was to construct Guttman-type scales with the ICF codes for use in geriatric care settings. The goal was to be able to use the scales to assign patients to one stage. Staging of the functional levels of patients enhances standardization of care, helps in the planning and development of health services, and allows for communication among health services professionals concerning patients’ functional capabilities. Therefore, we decided to construct a new ICF-based staging system, starting from ICF codes, rather than linkage from an extant measure, and to find a link to the ICF. This study departed from measurement of the ICF codes themselves. Using the results, we reconstructed a new measurement tool to stage the functioning of elderly persons.

## Methods

### Item selection and assessment

We selected 19 items related to mobility, walking, and transfer based on a previous study on reliability [4]. The 19 items were then modified into 33 items which specified performance in relation to mobility, walking and transfer. We divided the 33 ICF codes into 12 “basic mobility”-related items, and 21 “walking”-related items according to the meaning of each code. These modified items were labeled differently from the original items compared with the labeling used in the study. For example, the ICF code “Maintaining a standing position (d4154)” was divided into “Maintaining a standing position with assistance (d4154a)” and “Maintaining a standing position without assistance (d4154b).” The modified or specified ICF codes are shown in Additional file 1. All ICF items are attached with illustrations [21].

### Participants

In this study, we recruited two groups, one for scale development, and another for scale validation. For both groups, elderly persons over 65 years old were recruited. For scale development, Japanese elders from 14 institutions and 14 day care-services under the auspices of long-term care insurance (LTCI) were recruited. Each facility was asked to randomly select 10% of their users. The developmental sample was measured with the 33 ICF items.

For scale validation, data from 182 institutions and 177 day care centers were collected. Each facility randomly selected 10% of their patients for participation in the study. The ICF items selected by the scale development process and the newly constructed Guttmann-type scale were measured in this sample.

Each patient was measured with respect to each ICF item according to performance (whether or not the participants do a task as part of their daily activities) or capacity (whether or not the participants could do the task in a special or “standard” environment setting such as in a rehabilitation room). The performance results were used in this study. We did not use the 0-4 generic qualifier of the ICF. Each item was assessed/rated “yes” or “no” using the binary response options to construct a Guttmann-type scale.

The assessment was based on the observation of the daily activities in a geriatric health facility. For example, in the assessment of maintenance of sitting position, the authors did not specify the duration of maintenance of such activity unless specified otherwise, but if the elderly person was capable of maintaining a sitting position regularly, the assessor checked “yes” to this item.

The assessment was performed by trained health care professionals such as physiotherapists, occupational therapists, nurses, and certified nursing aides, who also had experience in geriatric health assessment. In addition, the health care professionals were given training by the authors on how to make the assessments using each ICF item.

Written consent to participate in this study was obtained from each participant or the participant’s proxy family member. The study was approved by the Ethical Review Board of the Japanese Association of Geriatric Health Services Facilities, and is in compliance with the Declaration of Helsinki.

### Data analysis

The characteristics of the sample and contrasts between the variables were analyzed using SPSS version 12.0 (IBM Corporation, Armonk, NY, USA). Rasch analysis was performed with RUMM2030 (RUMM Laboratory Pty. Ltd., Duncraig, WA, Australia). The Rasch model was employed to identify item-fitting and redundant items and to identify a hierarchy of mobility items ranked from easiest to hardest. For this analysis, a sample of 300 items was randomly selected from the eligible sample for scale development (n = 1164). If we use the total sample, most of the items appear to not fit the Rasch model because fit statistics are sample size dependent and as the sample here is relatively large. Therefore, all items would be significant and not fit the model unless a smaller random sample is selected [22]. Thus, taking into account the relationship between sample size and significance of mean-square statistics, the authors decided to use the sample size of 300 [23].

The ICF items that showed a low fit to the Rasch model were deleted iteratively until the remaining items reached an acceptable item fit (selection criteria P > 0.05). The iterative process is not shown in this paper. Of the items that showed a closer fit to the Rasch model, four items (out of 12 items) for basic mobility were selected, and four walking-related items (out of 21 items) were selected. A panel of health care professionals, including a physician, nurses, nursing aide, physiotherapist, and occupational therapist, reviewed the items selected after statistical selection of the items. If we had more than four items that fit the Rasch model, then the panel members chose the final item based on its applicability in daily care settings.

Then, two Guttman-type scales namely “basic mobility” and “walking” were constructed using the four ICF items selected for each scale, and illustrations were attached. Using the sample for scale validation, the threshold location of the newly developed scale was tested against the ICF items to see whether the order of the threshold was in order of difficulty for each ICF item. In addition, differential item functioning (DIF) for study location (day care and institution), sex and age-group (under 74 years, 75 to 84 years, 85 to 94 years and over 95 years) was tested for scale validation [24].

## Results

Among 1560 potential study candidates from the sample for scale development, 1164 were eligible for this study. A total of 396 participants were excluded due to missing data. Those persons with missing data did not differ significantly in terms of sex, age group and study location, according to the chi-square test. The average age of the candidates in the eligible sample was 84 (SD 8) years, and 222 (19%) of the study subjects were men. Of these, 313 (27%) elderly persons were living at home and assessed while using a day care service. The participants in the remaining sample were institutionalized elderly persons.

**Figure 1:**
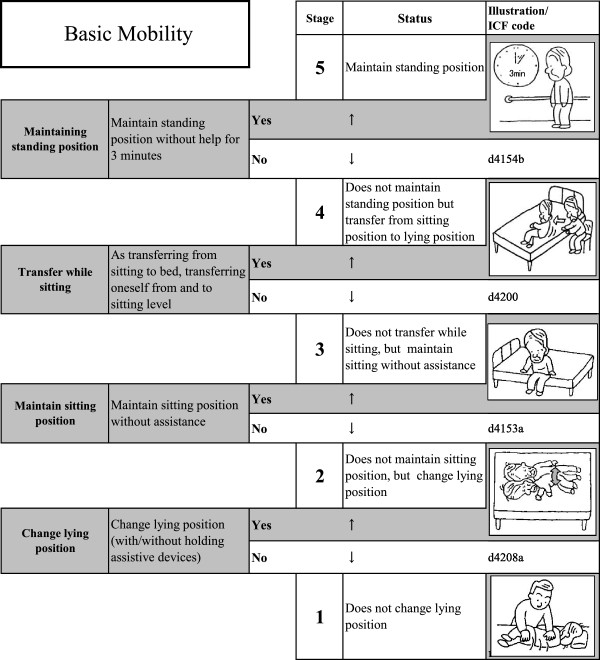
Basic mobility scale.

**Figure 2:**
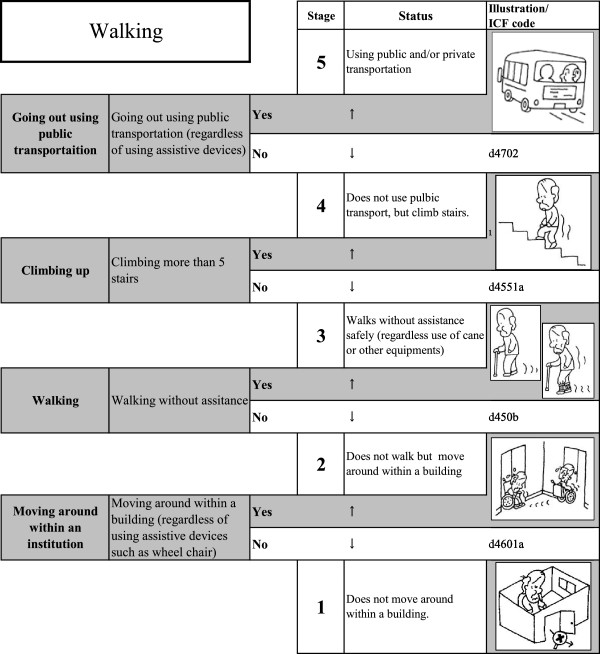
Walking scale.

Tables 1 and 2 show the location and fit statistics of the initial items tested. Of these, 12 items were selected which further described basic mobility. From Tables 1 and 2, we re-analyzed the remaining items until we selected the best items that fit the model. These items were then rearranged as a Guttman-type scale according to their item difficulty, as shown in Figures 1 (mobility scale) and Figure 2 (walking scale). Illustrations are attached to show the image for each ability level.

**Table 1:** Item locations and fit statistics for basic mobility

**ICF code**	**Item related to body movement and body posture**	**Location**	**Fitness**	**p-value**
d4100	Lying down	−1.53	4.87	0.09
d4103	Sitting	−0.86	3.56	0.17
d4105	Bending	−0.16	1.40	0.50
d4106	Shifting the body's center of gravity	0.80	9.16	0.01
d415	Maintaining a body position	−3.29	0.82	0.66
d4153a	Maintaining a sitting position without assistance	−0.33	3.33	0.19*
d4154a	Maintaining a standing position with assistance	1.84	3.48	0.18
d4154b	Maintaining a standing position without assistance	3.51	3.64	0.16*
d420	Transferring oneself	0.38	22.29	0.00
d4200	Transferring oneself while sitting	0.07	3.76	0.15*
d4201	Transferring oneself while lying	1.92	5.08	0.08
d4208a	Changing lying position	−2.35	1.50	0.47*

*final items selected.

**Table 2:** Item locations and fit statistics for walking functions

**ICF code**	**Item related to mobility and walking**	**Location**	**Fitness**	**p-value**
d450a	Walking with assistance from a person	−1.93	29.66	0.00
d450b	Walking without assistance	1.12	3.14	0.21*
d4500a	Walking short distances (50 m)	−0.88	4.50	0.11
d4500b	Walking short distances (50 m) on flat floor	−1.50	13.92	0.00
d4502	Walking on different surfaces	1.55	1.91	0.38
d4503	Walking around obstacles	−1.87	12.87	0.00
d4551a	Climbing (climbing upstairs)	1.64	0.52	0.77*
d4551b	Climbing (climbing downstairs)	1.22	2.78	0.25
d4601a	Moving around within buildings other than home (in nursing home)	−4.23	1.44	0.49*
d4601b	Moving around within buildings other than home (not nursing home)	−0.50	6.57	0.04
d4602	Moving around outside the home and other buildings	1.36	2.82	0.24
d465a	Moving around using equipment (with cane)	0.11	5.39	0.07
d465b	Moving around using equipment (with cane and braces)	1.04	3.49	0.17
d465c	Moving around using equipment (with T-shaped cane)	−0.18	3.70	0.16
d465e	Moving around using equipment (with four-point cane)	1.30	2.27	0.32
d465f	Moving around using equipment (with walker)	−0.43	3.67	0.16
d465g	Moving around using equipment (with circled-type walker)	1.55	2.05	0.36
d465h	Moving around using equipment (with wheelchair)	−2.42	187.31	0.00
d465i	Moving around using equipment (with braces)	0.98	4.41	0.11
d4701	Using private motorized transportation	−0.96	6.93	0.03
d4702	Using public motorized transportation	3.04	1.16	0.56*

*final items selected.

In the present study, the authors reduced the number of items by constructing Guttman scales in combination with Rasch analysis. An example of a basic mobility scale is shown in Figure 1. This Guttman-type scale is composed of 4 ICF items that were used as thresholds. The levels between the thresholds are labeled, and illustrations have been added to clarify each level. For example, as seen in Figure 1, stage 1 of the basic mobility scale is not being able to change in and out of a lying position independently. If the person is able to change position but does not maintain a sitting position, they are assessed as stage 2.

We tested the characteristics of the newly developed scale using the sample for scale validation. There were 1706 elderly persons using an institutional service (average age, 85 years) and 1554 elderly persons using a day care service (average age, 81 years) from whom we obtained the data for validation. There were more institutionalized elderly persons in the sample for scale validation, but the percentage according to sex did not differ significantly between the two groups. For the age category, the sample for validation was younger (average age, 82 years) compared with the sample for development (average age, 84 years) because the former included more elderly persons in the age group between ages 64 and 75 years.

**Figure 3:**
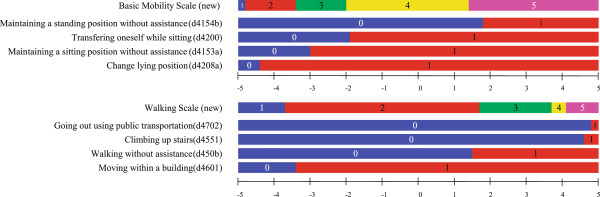
**Threshold distribution of new scales and original ICF items.** The borders of levels between 1, 2, 3, 4, 5 of the Basic Mobility and Walking Scale represent the locations of the threshold levels in logit. The border (0 and 1) of each ICF code represents the threshold of whether the person performed the task (=1) or not (=0).

Figure 3 shows the location of the ICF codes with respect to the new scale. The item difficulty (location) was found to be in the same order as the ICF codes selected to construct the items. No DIF was observed for study location (institution or day care), sex, or age groups (see Additional file 2).

## Discussion

The ICF-based classification developed in the present study has wide applicability. First, patients can be assigned to one stage in each scale. Staging offers standardization of rehabilitation and care management because patients of the same group in a certain level require a similar amount and type of resources. This was achieved by hierarchically rearranging the ICF items and constructing Guttman-type scales according to item difficulty.

This approach also provides the opportunity to analyze longitudinal changes in an elderly person’s functioning. Based on the results shown in Figure 3, the item location of each sample as shown was used to develop this scale. As shown here, patient characteristics are demarcated by ICF codes not only qualitatively, but also quantitatively. The location of each threshold item used to construct staging is arranged in a logit model. If a patient improves from one stage to the next stage, then the amount of improvement can be estimated by the difference between the two items’ locations. This means that the user can estimate patients’ functioning levels and follow them quantitatively.

Results from our study can also be used to allocate resources, such as for rehabilitation. Figure 3 shows the initial ICF items plotted on the new scale in order of item difficulty. As shown here, the patients within a specific category may or may not be able to perform the tasks represented by the adjacent ICF items. Therefore, these items can be used as proxy targets for rehabilitation.

Some ICF tools, such as ICF Core Sets, have used the ICF codes separately. ICF Core Sets have been developed in the effort to make the utility of the ICF practical and feasible, particularly in clinical settings. Only selected ICF categories that were found to be relevant to a specific health condition, setting, or context are included in a Core Set. Our approach differs from most ICF Core-Set approaches because our method does not select patients by diagnosis. This is because, in sub-acute care settings such as nursing homes or rehabilitation care facilities, as well as in home care settings such as day care, patients are not divided by disease category. Some cross-group difference was analyzed using DIF analysis and no DIF was found between the elderly persons in institutions and day-care facilities, which implies the applicability of this method for both settings. In addition, the scales can be used as a classification system because they have staging properties. By adding illustrations to the scales, a clear image concerning basic mobility and walking can now be obtained for each patient.

Our study does have some limitations. First, the location statistics of two ICF items used as thresholds, namely, ‘Going out using public transportation (d4702)’ and ‘Climbing up stairs (d4551),’ were very near each other, which results in weak discriminative power, as shown in Figure 3. This was also evident in the newly developed mobility and walking scale. ‘Going out using public transportation’ may require not only mobility functioning, but also adequate cognitive and orientation functioning. However, we retained this item in this scale, because for the elderly living at home, this skill is important for staying active in society. Second, we could not use the exact ICF codes because the ICF itself does not provide code definitions applicable or specifically intended for the geriatric setting. Therefore, we had to attach associated words to fit the geriatric care setting such as “Maintain sitting position without assistance” and “Walking without assistance”. Third, our study population is Japanese, which could limit the applicability of our findings to other types of patients, settings, geographical locations. Furthermore, our study was conducted in a government long-term care facility, which may impact the use of certain assessment instruments in other populations.

However, the scales we have developed satisfy content validity, because items were selected from a broad spectrum of mobility and walking. In addition, by dividing the items into the categories of basic mobility and walking, and allowing each item to have a closer fit to the Rasch model, the scales are likely to be both measuring a single dimension with different difficulty and satisfying the construct validity. Use of expert opinions to help selecting items for the scale also adds validity. However, further supporting evidence through subsequent studies will need to be considered.

The absence of differential item functioning across institutional and day care users, sex, and age group indicates the cross-group validity of the scale. Therefore, these scales may be ready for use as assessment scales in geriatric care settings. In addition, we can now better understand and manage patient care using functional information based on the ICF. As we used ICF as a basis for our taxonomy, these scales may be used internationally. However, the contextual difference in language across countries should be taken into account for international application.

Furthermore, aspects such as test-retest reliability and both concurrent and predictive validity are also essential elements of outcome measurement. Hence, these would need to be examined in future studies. Using the same methodology, ICF-based staging scales relating other aspects of ADLs, such as eating and toileting, as well as cognitive functioning and social participation, are under development.

## Conclusions

We have developed two simple staging scales for basic mobility and walking based on the ICF for elderly persons. Using these scales, patients are assigned to one stage in each scale. This was achieved by hierarchically rearranging the ICF items and constructing Guttman-type scales according to item difficulty using the Rasch model. These scales facilitate objective, simple and clear descriptions of elderly functional levels thereby improving the ability to use as a comparable assessment and staging tool. In addition, each functional level might require similar resources and therefore enable stan-dardization of care and rehabilitation. Illustrations facilitate the sharing of patient images among health care providers. The authors are currently performing additional validity and reliability studies to enable the scale to be used in international geriatric care settings.

## Competing interests

JO TT and TK are involved in the development of ICF-based care-management systems (R4 system). for the Japanese Association of Geriatric Health Services Facilities (JAGHSF) in which this ICF based staging will be used. JO TT and TK received travel expenses to develop this care-management system. However, JAGHSF is a not-for profit organization aiming at quality improvement of the member facilities. Therefore the authors declare no competing interests.

## Authors’ contributions

JO: conception and design, analysis and interpretation of data, preparation of the manuscript; TT and KT: acquisition and interpretation of data, revision of the manuscript; RE: interpretation of data, revision of the manuscript. The final version of the manuscript was approved by all authors.

## Pre-publication history

The pre-publication history for this paper can be accessed here:

http://www.biomedcentral.com/1471-2318/13/16/prepub

## Supplementary Material

Additional file 1 Items used in this study, with or without modification. Click here for file

Additional file 2 Differential item functioning for study location, sex and age-group. Click here for file

## References

[B1] World Health Organization International classification of functioning, disability and health: ICF 2001 Geneva: World Health Organization

[B2] Stucki G Cieza A Ewert T Kostanjsek N Chatterji S Ustün TB Application of the international classification of functioning, disability and health (ICF) in clinical practice Disabil Rehabil 2002 24 281 282 10.1080/09638280110105222 12004974

[B3] World Health Organization International Classification of Diseases Retrieved January 5, 2012, from http://www.who.int/classifications/icd/en/

[B4] Okochi J Utsunomiya S Takahashi T Health measurement using the ICF: test-retest reliability study of ICF codes and qualifiers in geriatric care Health Qual Life Outcomes 2005 3 46 10.1186/1477-7525-3-46 16050960 PMC1199614

[B5] Jette AM Norweg A Haley SM Achieving meaningful measurements of ICF concepts Disabil Rehabil 2008 30 963 969 10.1080/09638280701800426 18484391

[B6] Dreinhöfer K Stucki G Ewert T Huber E Ebenbichler G Gutenbrunner C Kostanjsek N Cieza A ICF core sets for osteoarthritis J Rehabil Med 2004 44 75 80 15370752 10.1080/16501960410015498

[B7] Geyh S Cieza A Schouten J Dickson H Frommelt P Omar Z Kostanjsek N Ring H Stucki G ICF core sets for stroke J Rehabil Med 2004 44 135 141 15370761 10.1080/16501960410016776

[B8] Stucki G Grimby G ICF core sets for chronic conditions 2004 Abingdon, Oxon, UK: Taylor & Francis

[B9] World Health Organization ICF Checklist version 2.1a, clinician form Retrieved January 5, 2012, from http://www.who.int/classifications/icf/training/icfchecklist.pdf

[B10] Stucki G Ewert T Cieza A Value and application of the ICF in rehabilitation medicine Disabil Rehabil 2002 24 932 938 10.1080/09638280210148594 12523361

[B11] Granlund M Eriksson L Ylvén R Utility of international classification of functioning, disability and health's participation dimension in assigning ICF codes to items from extant rating instruments J Rehabil Med 2004 36 130 137 10.1080/16501970310021707 15209456

[B12] Alviar MJ Olver J Brand C Hale T Khan F Do patient-reported outcome measures used in assessing outcomes in rehabilitation after hip and knee arthroplasty capture issues relevant to patients? results of a systematic review and ICF linking process J Rehabil Med 2011 43 374 381 10.2340/16501977-0801 21448553

[B13] Muller M Stier-Jarmer M Quittan M Strobl R Stucki G Grill E Validation of the comprehensive ICF Core Sets for patients in early post-acute rehabilitation facilities J Rehabil Med 2011 43 102 112 10.2340/16501977-0659 21234511

[B14] Granger CV Deutsch A Linn RT Rasch analysis of the functional independence measure (FIM) mastery test Arch Phys Med Rehabil 1998 79 52 57 10.1016/S0003-9993(98)90208-8 9440418

[B15] Cieza A Hilfiker R Boonen A van der Heijde D Braun J Stucki G Towards an ICF-based clinical measure of functioning in people with ankylosing spondylitis: a methodological exploration Disabil Rehabil 2009 31 528 537 10.1080/09638280802173475 18608418

[B16] Cieza A Hilfiker R Chatterji S Kostanjsek N Ustün BT Stucki G The international classification of functioning, disability, and health could be used to measure functioning J Clin Epidemiol 2009 62 899 911 10.1016/j.jclinepi.2009.01.019 19540718

[B17] Pallant JF Keenan AM Misajon R Conaghan PG Tennant A Measuring the impact and distress of osteoarthritis from the patients' perspective Health Qual Life Outcomes 2009 7 37 10.1186/1477-7525-7-37 19400966 PMC2683800

[B18] Okochi J Takahashi T Takamuku K Matsuda S Takagi Y Reliability of a geriatric assessment instrument with illustrations Geriatr Gerontol Int 2005 5 37 47 10.1111/j.1447-0594.2005.00268.x

[B19] Okochi J Takahashi T Kroll T Application of the ICF codes in geriatric assessment: use of the ICF qualifiers to quantify health information Focus on disability: trends in research and application, Volume Volume II 2007 Central City, CO, U.S.A: Nova 39 56

[B20] Küçükdeveci AA Sahin H Ataman S Griffiths B Tennant A Issues in cross-cultural validity: example from the adaptation, reliability, and validity testing of a Turkish version of the Stanford health assessment questionnaire Arthritis Rheum 2004 51 14 19 10.1002/art.20091 14872450

[B21] Sutch S ICF illustration library Bull World Health Organ 2004 82 550 551

[B22] Smith R Schumacker RE Bush M Using item mean squares to evaluate fit to the Rasch model J Outcome Meas 1998 2 66 78 9661732

[B23] Institute for Objective Measurement Rasch Power Analysis: Size vs. Significance: Infit and Outfit Mean-Square and Standardized Chi-Square Fit Statistic Retrieved January 5, 2012, from http://www.rasch.org/rmt/rmt171n.htm

[B24] Bond T Cristin M Bond T, Cristin M Rasch model applied: rating scale design Applying the Rasch model 2001 New Jersey: Lawrence Erlbaum Associates, Inc 158 172

