# Supplementary material for: Staging of mobility, transfer and walking functions of elderly persons based on the codes of the International Classification of Functioning, Disability and Health
Source: BMC Geriatr. 2013 Feb 15;13:16. doi: 10.1186/1471-2318-13-16 (PMC3598939; doi:10.1186/1471-2318-13-16)
Supplement: Additional file 2 — Differential item functioning for study location, sex and age-group. [file 1471-2318-13-16-S2.doc]

**Appendix 2**

**Plot of differential item functioning of Basic Mobility Scale and Walking Scale**

**Basic Mobility Scale**

**Study location**

**
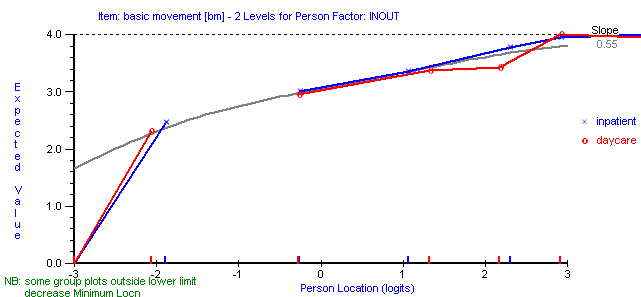
**

**Sex**

**
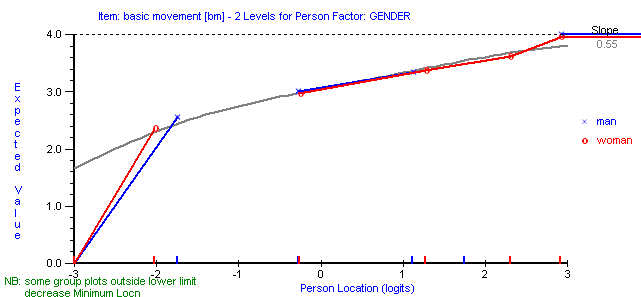
**

**Age group**

**
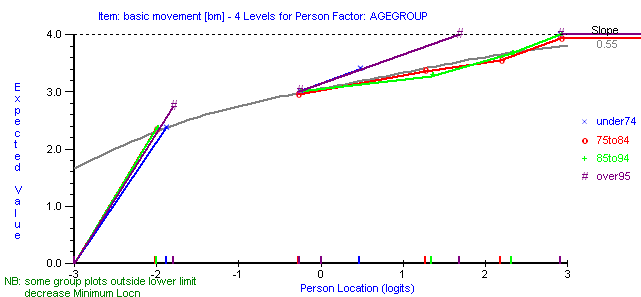
**

**Walking Scale**

**Study location**

**
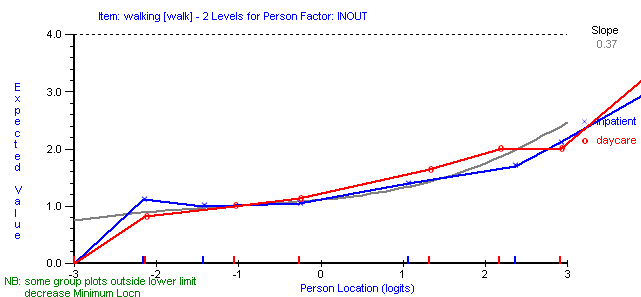
**

**Sex**

**
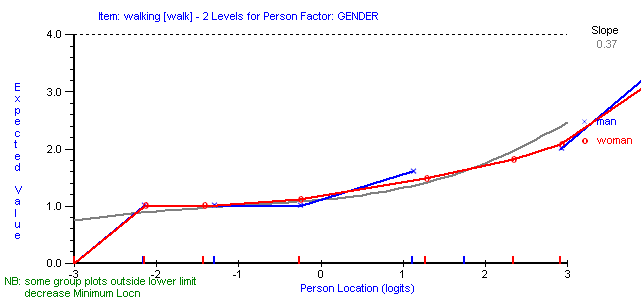
**

**Age group**

**
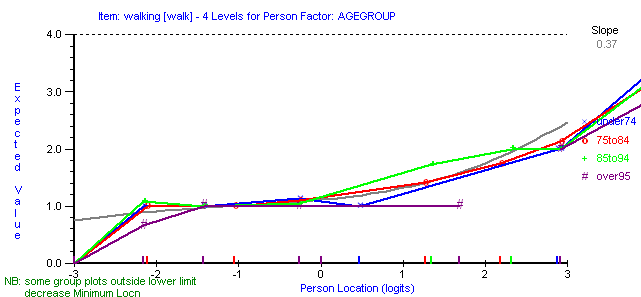
**
